# The role of circulating miRNAs and CA19-9 in pancreatic cancer diagnosis

**DOI:** 10.18632/oncotarget.28038

**Published:** 2021-08-17

**Authors:** Nivaldo Faria Vieira, Luciano Neder Serafini, Paulo Cezar Novais, Fermino Sanches Lizarte Neto, Mucio Luiz de Assis Cirino, Rafael Kemp, José Celso Ardengh, Fabiano Pinto Saggioro, Alberto Facury Gaspar, Ajith Kumar Sankarankutty, Jorge Resende Lopes Júnior, Daniela Pretti da Cunha Tirapelli, José Sebastião dos Santos

**Affiliations:** ^1^Department of Surgery and Anatomy, Medical School of Ribeirão Preto, University of São Paulo, Ribeirão Preto, São Paulo, Brazil; ^2^Department of Pathology, Medical School of Ribeirão Preto, University of São Paulo, Ribeirão Preto, São Paulo, Brazil; ^3^Postgraduate Program in Structural and Functional Interactions in Rehabilitation, University of Marilia, Marília, Brazil; ^4^Clinical Hospital of the Medical School of Ribeirão Preto, University of São Paulo, São Paulo, Brazil

**Keywords:** pancreatic cancer, microRNA, CA19-9, biomarkers, cancer recurrence

## Abstract

Diagnosis and treatment of pancreatic ductal adenocarcinoma (PA) remains a challenge in clinical practice. The aim of this study was to assess the role of microRNAs (miRNAs-21, -23a, -100, -107, -181c, -210) in plasma and tissue as possible biomarkers in the diagnosis of PA. Samples of plasma (PAp-n = 13), pancreatic tumors (PAt-n = 18), peritumoral regions (PPT-n = 9) were collected from patients during the surgical procedure. The control group consisted of samples from patients submitted to pancreatic surgery for trauma or cadaveric organs (PC-n = 7) and healthy volunteers donated blood (PCp-n = 6). The expression profile of microRNAs was measured in all groups using RT-PCR, serum CA19-9 levels were determined in PA and PC. In tissue samples, there was a difference in the expression of miRNAs-21, -210 (*p* < 0.05) across the PAt, PC and PPT groups. The PAp showed overexpression of miRNAs-181c, -210 (*p* < 0.05) when compared to PCp. The combination of miRNAs-21, -210 tissue expression and serum CA19-9 showed 100% accuracy in the diagnosis of PA, as well as miR-181c expression in the plasma (PApxPCp). The expression of microRNAs in plasma proved to be a promising tool for a noninvasive detection test for PA, as well as further studies will evaluate the utility of microRNAs expression as biomarkers for prognostic and response to therapy in PA.

## INTRODUCTION

Pancreatic ductal adenocarcinoma (PA) represents only 2.8% of all new cases of cancer in the US [1]. However, PA is the fourth leading cause of cancer deaths and less than 5% of patients will survive for 5 years [2]. Thus, the identification of noninvasive methods for screening, diagnosis, staging and follow-up for different therapeutic options must be investigated.

MicroRNAs (miRNAs) are a class of small noncoding RNAs consisting of 18–25 nucleotides that function by targeting specific mRNA for translational repression or degradation, thereby regulating several biological processes including cell proliferation, migration, invasion, survival, and metastasis. They act as negative regulators of the protein-coding gene expression by targeting mRNA [3] and due to their biological stability and role in cancer pathobiology, miRNAs have a substantial potential as cancer biomarkers.

Multiple biological processes can be regulated by miRNAs and since the first miRNA was identified in pancreatic tissue [4], several miRNAs have been found to be involved in pancreatic oncogenesis [5]. The most important oncogenic miRNAs for PA are miR-21 [6], miR-155 [7], miR-107 [8], miR-210 [9], miR-23a [10], miR-373 [11], miR-221/222 [12], and miR-181 [13]. Also, let-7a [14], miR-96 [15], miR-375 [16], miR-20a [17], and miR-200c [18] act as tumor suppressor miRNAs.

Plasma cell-free miRNAs have been pointed out as important future clinical biomarkers in different tumors [19–21]. Several miRNAs may have a role as pancreatic cancer biomarkers, such as miR-21 [12], miR-155 [12], miR-210 [22], miR-1290 [23], miR-22 [24]. MiRNAs can also regulate the response of tumor cells to chemotherapeutic agents, as many studies have proven in recent years [25].

The present study aimed to assess the utility of selected miRNAs in plasma and pancreatic tissue as diagnostic markers for differentiating PA patients from individuals with no pancreatic disorders.

## RESULTS

### Study population

The study included a total of 31 individuals identified in the Biological Repository, 18 of them with PA (PAt group), 7 assigned to the tissue control group (PC Group) and 6 assigned to the plasma control group (PCp Group). The characteristics of the subjects are summarized in Table 1.

**Table 1:** Patient characteristics

Variable		PA	Tissue Control	Plasma Control
		*n* = 18	*n* = 7	*n* = 6
**Gender**	Male	10	4	3
	Female	8	3	3
**Age**	<60	3	5	4
	>60	15	2	2
**Pathology**				
**Stage**	<T3	9	-	-
	T3–T4	9	-	-
	Regional Lymph node	4	-	-
	Metastasis	11	-	-
**TNM**	I	1	-	-
	IIa	2	-	-
	IIb	2	-	-
	III	2	-	-
	IV	11	--	-
**Family history of cancer**	Any relative	7	-	-
**Weight loss**	>5 kg	12	-	-
	≤5 kg	6	-	-
**Jaundice**	Present	12	-	-
**Surgery**	Duodenopancreatectomy	6	-	-
	Biliodigestive Anastomosis	10	-	-
	Exploratory Laparotomy	2	-	-
**Chemotherapy**	Adjuvant	2/6	-	-
	Palliative	8/12	-	-

### miRNAs expression in tissue

**Figure 1:**
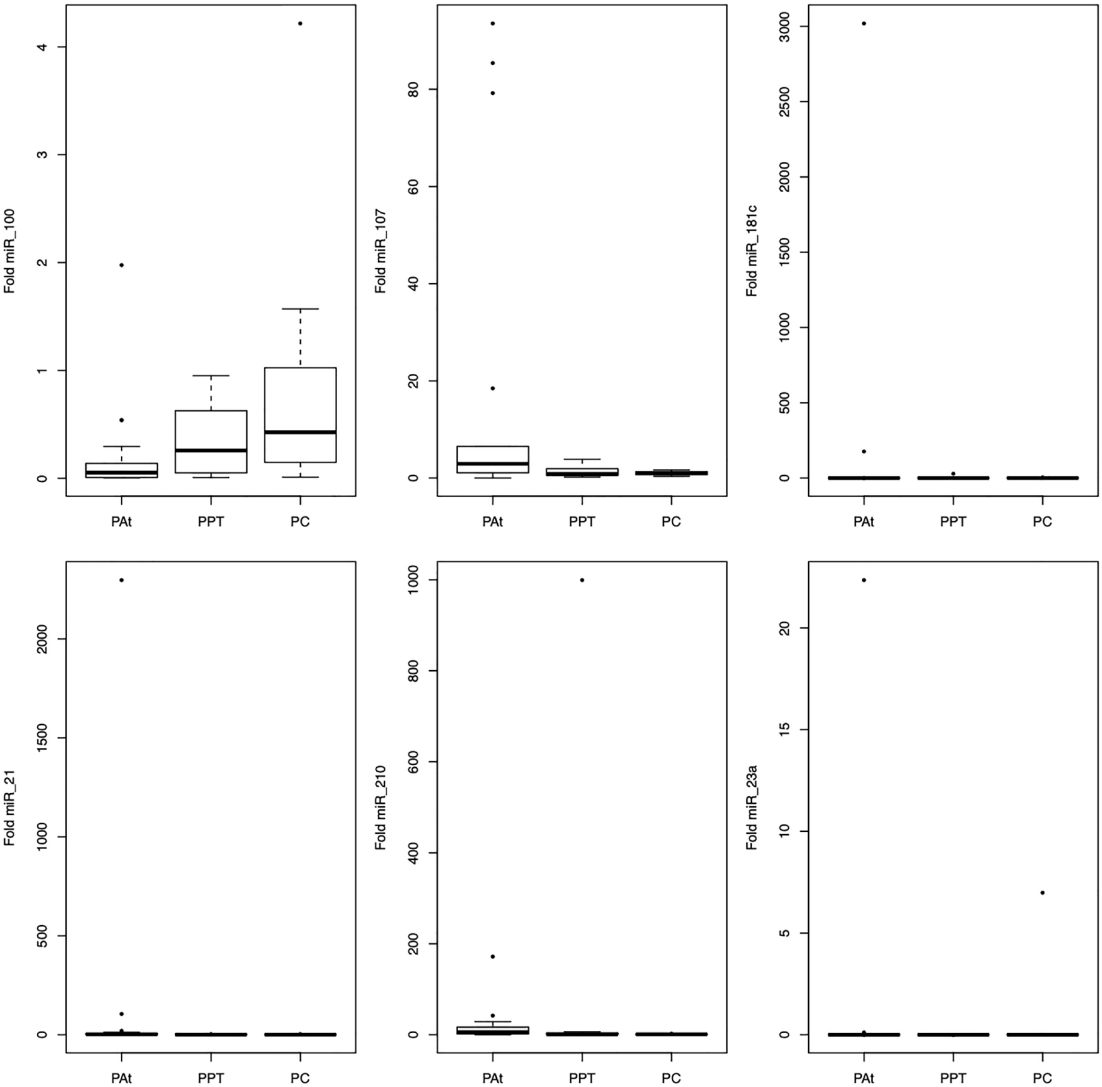
Representation of the average values of the miRNAs-100, -107, -181c, -21, -210, -23a expression in the tissue among the studied groups.

We evaluated six miRNAs in PA tissue (PAt), peritumoral tissue (PPT), and control normal pancreatic tissue (PC). There were differences in miRNA-21 (2.4) expression between groups PAt, PPT (0.86) and PC (0.87) (*p* = 0.005), and in miRNA-210 (5.43) expression between groups PAt, PPT (0.77) and PC (0.99) (*p* = 0.008) (Figure 1).

Comparative analysis using the Dunn post-test showed that miRNA-21 discriminated between the PAt and the PC and PPT groups and miRNA-210 discriminated between the PAt and PC groups (Table 2).

**Table 2:** Statistical analysis with critical values for the discrimination between the PAt group and the PC and PPT groups

	Observed difference	Critical difference	Difference
**miR-21**			
PAt – PPT	10.08	9.73	True
PAt - PC	11.99	10.61	True
PPT – PC	1.91	12.01	False
**miR-210**			
PAt – PPT	7.47	9.73	False
PAt - PC	12.92	10.61	True
PPT- PC	5.45	12.01	False

Dunn post test (95%).

### miRNAs expression in plasma

**Figure 2:**
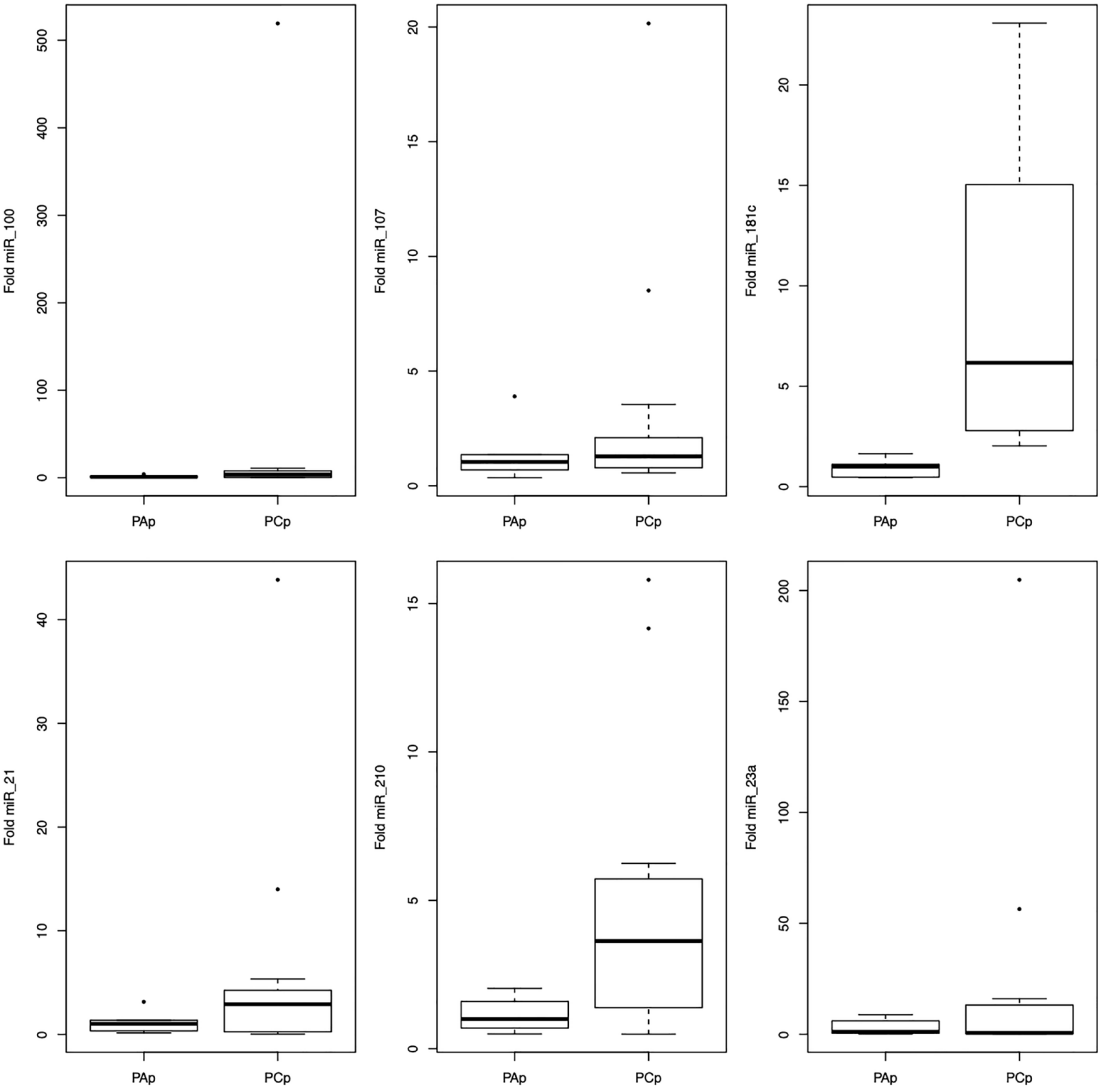
Representation of the average values of the miRNAs-100, -107, -181c, -21, -210, -23a expression in the plasma among the studied groups.

Only two miRNAs, miR-181c and miR-210, showed deregulation in plasma analyses (Figure 2). Both miR-181c (95% CI1.67–17.68; *p* < 0.0001) and miR-210 (95% CI 0.16–5.20; *p* = 0.03) were overexpressed in the PAp group compared to the PCp group.

### miRNAs as biomarkers

To determine if these tissue or plasma miRNAs could help to discriminate between patients and controls, we performed sensitivity, specificity, accuracy, and ROC curve analyses (Tables 3 and 4). Regarding tissue miRNA expression, miR-21 (76%; cut-off 1.03) and miR-210 (76%; cut-off 2.44) showed the best accuracy in distinguishing patients from controls.

**Table 3:** ROC analysis of the relative abundance of selected miRNAs

miRNA	Comparative	AUC	95% Confidence Interval	
			Lower Limit	Upper Limit
**miR-21**	^*^PAt × PC	**0.85**	**0.65**	**0.98**
	^*^PAt × PPT	**0.80**	**0.62**	**0.94**
	PPT × PC	0.42	0.14	0.74
	PAp × PCp	0.65	0.38	0.88
**miR-23a**	PAt × PC	0.69	0.44	0.92
	PAt × PPT	0.64	0.42	0.85
	PPT × PC	0.60	0.30	0.88
	PAp × PCp	0.47	0.23	0.74
**miR-100**	PAt × PC	0.73	0.48	0.92
	PAt × PPT	0.70	0.47	0.89
	PPT × PC	0.59	0.27	0.87
	PAp × PCp	0.61	0.36	0.85
**miR-107**	PAt × PC	0.75	0.54	0.92
	PAt × PPT	0.71	0.50	0.89
	PPT × PC	0.52	0.24	0.84
	PAt × PC	0.64	0.33	0.92
**miR-181c**	PAt × PC	0.72	0.52	0.90
	PAt × PPT	0.49	0.26	0.71
	PPT × PC	0.65	0.36	0.92
	^*^PAp × PCp	**1**	**1**	**1**
**miR-210**	^*^PAt × PC	**0.88**	**0.73**	**1**
	PAt × PPT	0.72	0.47	0.93
	PPT × PC	0.35	0.09	0.67
	^*^PAp × PCp	**0.80**	**0.59**	**0.97**

^*^Comparisons with AUC ≥ 0.80 considered to indicate moderate to strong association by the ROC method.

**Table 4:** Comparison of plasma miRNAs expression between groups PAp and PCp

Test	Cut	Sensibility	Specificity	Positive LR	Negative LR	Accuracy
miRNA-100	1.79	0.61 (0.32; 0.84)	0.83 (0.36; 0.99)	3.69 (0.58; 23.25)	0.46 (0.21; 1.00)	68%
miRNA-107	0.76	0.92 (0.62; 0.99)	0.50 (0.13; 0.86)	1.84 (0.81; 4.17)	0.15 (0.01; 1.19)	79%
**miRNA-181c**	**2.03**	**1.00 (0.71; 0.99)**	**1.00 (0.51; 0.99)**	**-**	**0**	**100%**
miRNA-21	1.35	0.61 (0.32; 0.84)	0.66 (0.24; 0.94)	1.84 (0.55; 6.19)	0.57 (0.23; 1.40)	63%
miRNA-210	1.98	0.69 (0.38; 0.89)	0.83 (0.36; 0.99)	4.15 (0.66; 25.77)	0.36 (0.15; 0.89)	74%
miRNA-23a	0.59	0.46 (0.20; 0.73)	0.33 (0.05; 0.76)	0.69 (0.30; 1.56)	1.61 (0.46; 5.57)	42%

Regarding to plasma miRNA expression, miR-181c (virtually 100%; cut-off 2.03), miR-107 (79%; cut-off 0.76) and miR-210 (74%; cut-off 1.98) showed the best accuracy in distinguishing patients from controls (Table 4).

**Figure 3:**
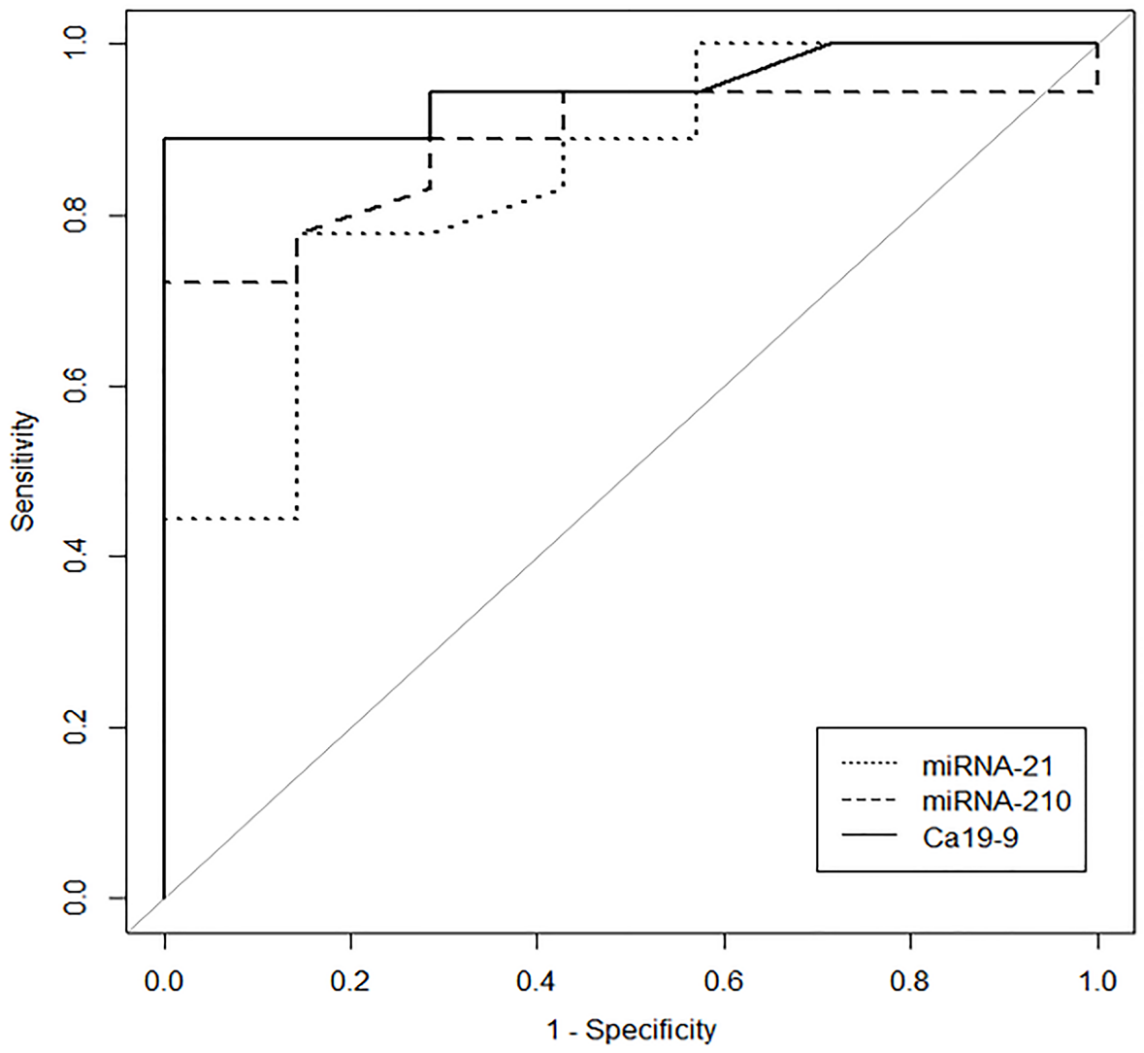
The ROC Curve illustrates the performance of tissue miR-21, tissue miR-210 and plasma CA 19-9 for group PAt x group PC distinguishing individuals with pancreatic cancer.

**Figure 4:**
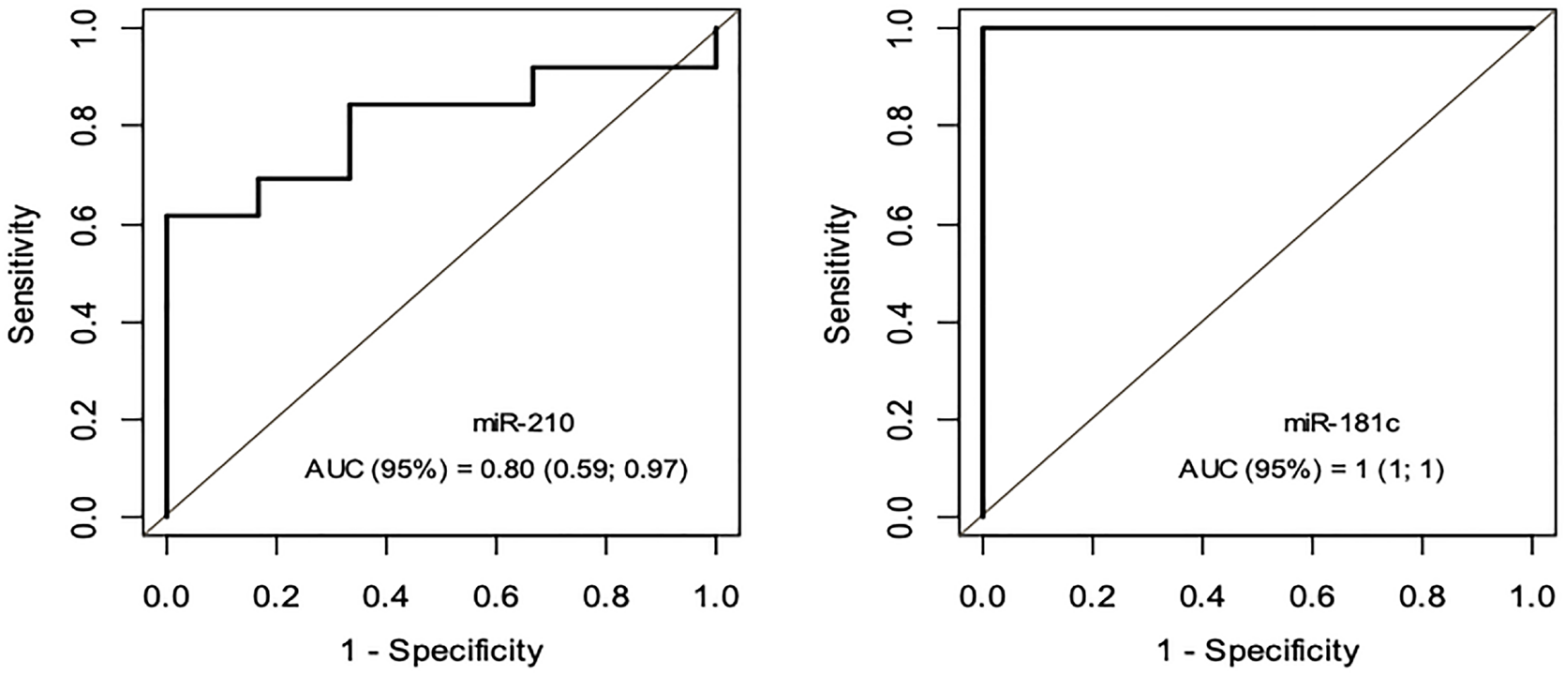
ROC Curve - miRNA-181c e miRNA-210 – Grupo PAp x Grupo PCp.

A ROC AUC above 0.80 is recommended for clinical applications. Two tissue miRNAs had an AUC above 0.80: miR-21 (0.85) and miR-210 (0.88) (Figure 3). Three other miRNAs had worthless AUC values: miR-107 (0.75), miR-100 (0.73) and miR-181c (0.72). Only two plasma miRNAs had great AUC values: miR-181c (virtually 1) and miR-210 (0.80) (Figure 4).

**Figure 5:**
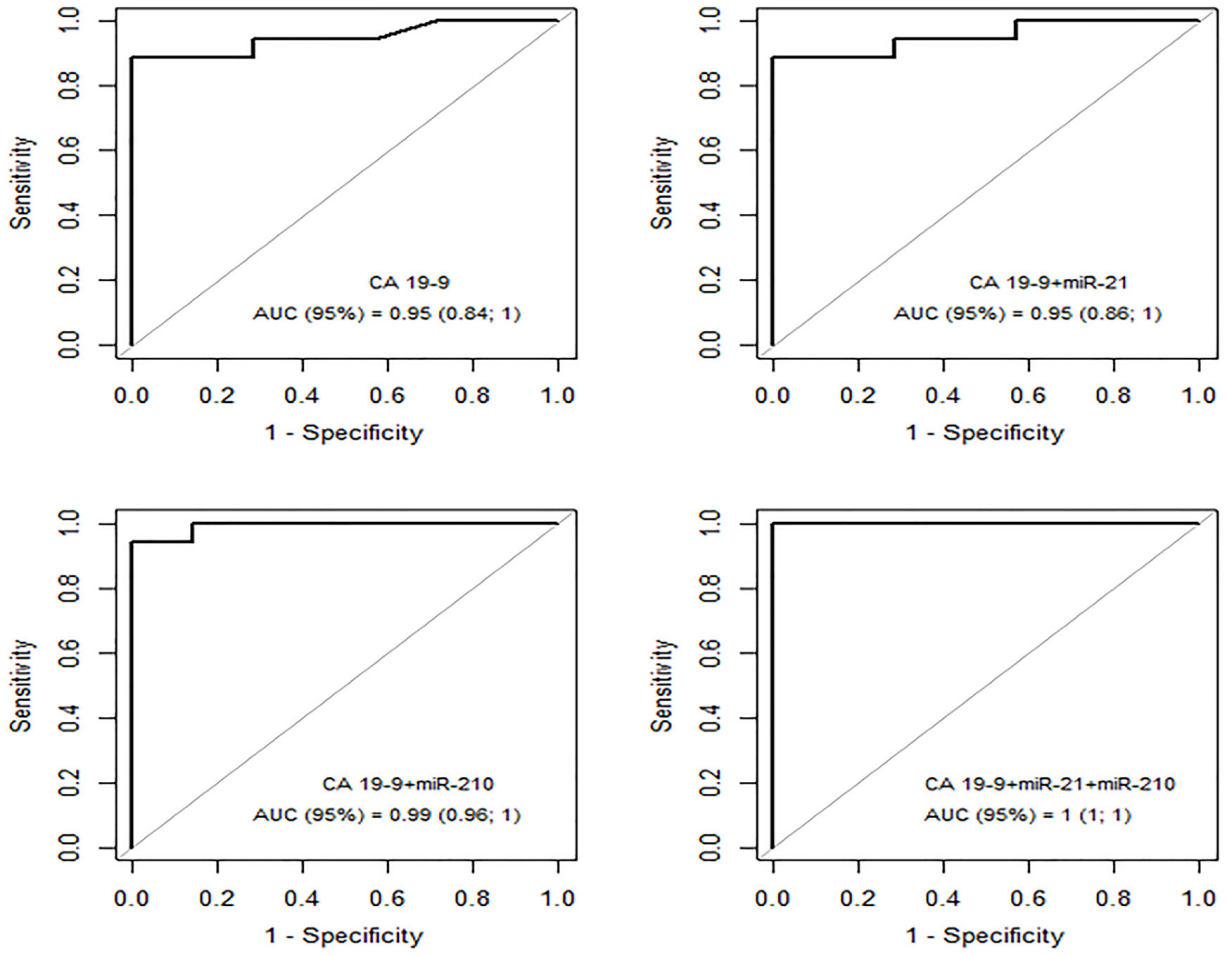
ROC curve – CA19-9; CA19-9 + miRNA21; CA 19-9 + miRNA210 and CA19-9+miRNA21+miRNA210 for group PAt x group PC.

CA19-9 showed an AUC of 0.95 in these patients. Combining miR-210 with CA19-9 increased the AUC to 0.99, but miR-21 did not have the same effect. However, the combination of CA19-9 with miRNA21 and miRNA210 increased the ROC-AUC to 1 (95% CI, 1–1), with 100% sensitivity, 100% specificity and a cut-off of 13.22 (Figure 5).

## DISCUSSION

PA accounts for 95% of all pancreatic cancers and only 6% of patients will survive for 5 years [26, 27]. Given these dismal statistics, there is substantial interest in developing novel tests to identify PA at an earlier stage or even in precursor lesions such as pancreatic intraepithelial neoplasia or early-stage intraductal papillary mucinous neoplasm [28–30].

In addition, a peripheral biomarker can be used as a first-line test for patients who have obstructive jaundice and suspected PA, as well as to guide response to modalities treatment options available.

MiRNAs, which are short non-coding RNAs, represent an attractive class of diagnostic and prognostic biomarkers for clinical application as they remain stable in tissue and bodily fluids and influence the pathobiology of cancer cells by altering the expression of several proteins such as epidermal growth factor receptor. Case–control studies evaluating different miRNA profiles in whole blood [12, 31] or plasma/serum [5, 20, 32, 33] have yielded varying results.

Most patients included in this study (11–61%) already had metastases, which influenced the selection of miRNA associated with this condition and the poor prognosis. There are review and meta-analysis studies demonstrating the association of increased miR-21 expression in pancreatic cancer with worsening prognosis of the disease [34–36].

Like miR-21, overexpression of miR-100 is associated with disease progression and poorer overall survival [37]. miR-181c, upon microarray analysis was significantly increased in 136 patients with pancreatic cancer and higher levels of its expression are related to more advanced tumor stages and lower survival rate [38]. Overexpression of miRNAs-21, -23a and -27a is associated with lower survival after pancreatectomy. These three miRNAs can act synergistically to inhibit the tumor suppressor pathway related to PDCD4, BTG2, and NEDD4L [10].

A significant correlation was observed between high expression levels of miRNAs-210, -155, -203 and -222 and reduced survival of patients with pancreatic tumors by up to 6.2 times [9]. The miR-107 in pancreatic cancer is associated with increased incidence and poor prognosis and may be involved in the possibility of metastasis and invasion through the TGFBR3 regulatory pathway [39].

In the present study, overexpression of miR-21, miR-107 and miR-210 was demonstrated in pancreatic cancer tissue compared to normal pancreatic tissue. Furthermore, in plasma, miR-181c and miR-210 were overexpressed in PA patients compared to controls. miR-181c was the only isolated miRNA whose expression was better than CA19-9 for distinguishing patients from controls, suggesting a role for plasmatic expression of miR-181c as a potential biomarker for PA.

The findings of the present study reproduce the PA tissue overexpression of miR-21 [6], miR-107 [8], and miR-210 [9] and hypoexpression of miR-100 [33]. However, in contrast to a previous study, miR-23a did not discriminate patients with PA from healthy ones through tissue and plasma. The previous study performed microarray analysis [10, 12] whereas we used qPCR. In addition, variations in gene expression between different populations have already been shown [40, 41] and can explain this difference.

In the tissue, miR-21 showed 76% accuracy in discriminating PA patients, although its overexpression was not observed in plasma, which contrasts with a study that detected a 2 to 20x increase in this miRNA [12]. These dissimilarities may be attributed to differences in the number of patients and in the studied populations as well as differences in disease staging, ethnic and geographic differences, among others.

There are studies that associate the expression profile of the studied miRNAs with chronic pancreatitis. However, the issue is very pertinent, since changes in pancreatic tissue perfusion, with repercussions on the epithelial-mesenchymal transition and on signaling pathways, regulate the inflammation and fibrosis present in chronic pancreatitis and pancreatic adenocarcinoma, may be associated with investigated miRNAs, and consequently, reduce the possibility of discrimination between the two clinical conditions.

Despite the absence of established links between CP and miRNAs, serum miRNAs associated with cancer and the inflammatory process were assessed aiming to be used to differentiate between CP and PA, specifically miR-10b-5p, miR-106b-5p, miR-210-3p and miR-216a-5p, all of which have been previously found to be significantly upregulated in PC.

Amongst the selected miRNAs, miR-210-3p was deemed to be the most promising serum biomarker to differentiate between CP and PA, as its expression was higher in patients with PA compared with those with CP, miRNA-106b-5p expression tended to be higher in patients with PA than those with CP. Comparative analysis showed significantly higher expression levels of miR-210-3p in patients with PA compared with patients with CP (*P* = 0.015), whereas expression of miR-106b-5p and miR-10b-5p tended to be higher in the patients with PA compared with those with CP, although the difference was not significant (*P* = 0.056 and *P* = 0.080, respectively) [42].

In other studies, the expression levels of the miRNAs-21 and -210 were also significantly higher in the serum of patients with PA compared to patients with chronic pancreatitis and healthy individuals [42, 43].

In the study of tumor tissue samples, chronic pancreatitis and normal adjacent tissue, was observed the hyperexpression of miR-21, miR-34a and miR-198 and reduced expression of miR-217 in PA compared to CP and normal tissue. Using ROC analysis, miR-21 was shown to have the highest capacity to distinguish between PA and CP with the sensitivity 93%, specificity 72% and AUC = 0.9227 [44], also there are miRNAs in the blood capable of differentiating patients with PA or CP and normal individuals. 100 miRNAS in tissue samples allowed to differentiate patients with PA and CP. Six miRNAs (miR-7, miR-151-3p, miR-194, miR-486-5p, miR-514 and miR-1206) were discriminating for CP and normal tissue [45].

Overexpression of 16, 14 and 9 miRNAs in PA, IPMN and CP, respectively, was demonstrated by comparison with plasma control. The miR-21-5p, miR-33a-3p, miR-320a, and miR-93-5p showed greater ability to discriminate for pancreatic neoplasia (PDAC and IPMN). Among them, there were 9 miRNAs (miR-151b, miR-16-5p, miR-181a-5p, miR-192-5p, miR21-5p, miR-320a, miR-33a-3p, miR-548d-3p, and miR-93-5p) with also significant higher levels in CP than in plasma control [46].

In this context, the miRNAs selected in this study and overexpressed in PA also need to be studied in CP. In the literature, miRNAS from families 21 and 181 (miR-21, miR21-5p and -181a-5p) have significantly higher levels in CP than in plasma control.

Regarding plasma analysis, although we did not find differences in miR-21 expression, we detected miR-210 overexpression in PA patients compared to controls. miR-21 has already been validated by qPCR [6] and this difference can be explained by sample size and ethnic population. Nevertheless, miR-210 was overexpressed in both plasma and tumor tissue in PA patients, as previously reported [22]. Also, comparison of the tumoral and peritumoral regions revealed miR-210 overexpression, suggesting that the increase of this miRNA originates from a reaction of the tumor-paratumor microenvironment. Most interesting is the fact that this is the first time that miR-181c overexpression is reported in the plasma of PA patients.

miR-181c overexpression has been shown in gastric cancer [47] and is reduced in cervical squamous cell carcinoma after surgery [48]. The oncogenic role of miR-181c is not totally understood, with this miRNA being overexpressed in primary neuroblastomas and reduced in metastatic lesions [49]. In neuroblastoma cells, miR-181c seems to inhibit cell proliferation, migration, and invasion [49]. Since we did not detect overexpression of miR-181c in tumor tissue samples, its plasma overexpression can be explained as a microenvironment negative response to an aggressive tumor. Thus, we may speculate that the expression of this miRNA is associated with the cancer stem cell subpopulation, currently implicated in resistance to treatment, relapse and metastasis [50].

CA19-9 is the most frequently used marker in clinical practice, with 70–80% sensitivity but with specificity of less than 50% for PA patients with values of 40 U/ml [51]. A limitation of CA19-9 is its reduced specificity in the setting of obstructive jaundice; the miRNAs studied here were unaffected by the presence of jaundice.

Serum CA19-9 concentration in the group of patients with PA showed 89% sensitivity and 100% specificity for a value of 16.20 U/l, with 88% accuracy, which was exceeded only by serum expression of miR-181c, with 100% sensitivity, specificity and accuracy for a value of 2.03. This is a promising result that opens the possibility of studies of miR-181c as an effective and less invasive biomarker. However, this result should be considered with caution in view of the small sample studied which consisted of 67% of patients with an advanced stage of PA.

Regardless of its function, miR-181c seems to be better than CA19-9 in discriminating between PA patients and controls. This is not the first time that miRNAs were compared to CA19-9, and a large study recently showed that a panel of miRNAs can add discriminating power to CA19-9 [52].

In the present study, when CA19-9 concentration was combined with the overexpressed miRNAs (miR-21 and -210), there was a significant increase in accuracy, with this combination possibly being of value for the differential diagnosis of PA.

Under the study conditions, overexpression of miR-21 and miR-210 was demonstrated in PA tissue compared to normal pancreatic tissue. In addition, plasma expression of miR-181c and miR-210 was found to be overexpressed in patients with PA compared to normal controls. Most interestingly, in plasma, miR-181c was found to be a better biomarker than CA19-9 for distinguishing PA patients from controls. Prospective studies, including patients with non-cancer pancreatic disease will be necessary to confirm the role of miR-181c as a PA biomarker.

The present findings reveal that the combination of miR-21 and -210 expression in tumor tissue with the CA19-9 values increases the accuracy of the PA diagnosis, while the expression of miR-181c in plasma may represent a noninvasive diagnostic test for the disease. Further studies are needed to validate the diagnostic accuracy of miR-181c in the plasma and to evaluate its utility as a biomarker of PA.

## MATERIALS AND METHODS

### Study design

A retrospective case-control pilot study was conducted in the Department of Surgery of the University Hospital, School of Medicine of Ribeirão Preto, University of São Paulo, a tertiary referral center in Brazil. The study was approved by the local Institutional Review Board (Process no 4910/2008).

### Subjects

We selected patients from a cohort who had been prospectively enrolled between April 2008 and August 2012 into a Pancreatobiliary Diseases Database and Biological Repository at the Ribeirao Preto School of Medicine, University of São Paulo. This database includes individuals undergoing procedures in the pancreas and biliary tract both for malignant and benign diseases. From this Repository, we selected patients with available pancreatic tissue and plasma.

**Figure 6:**
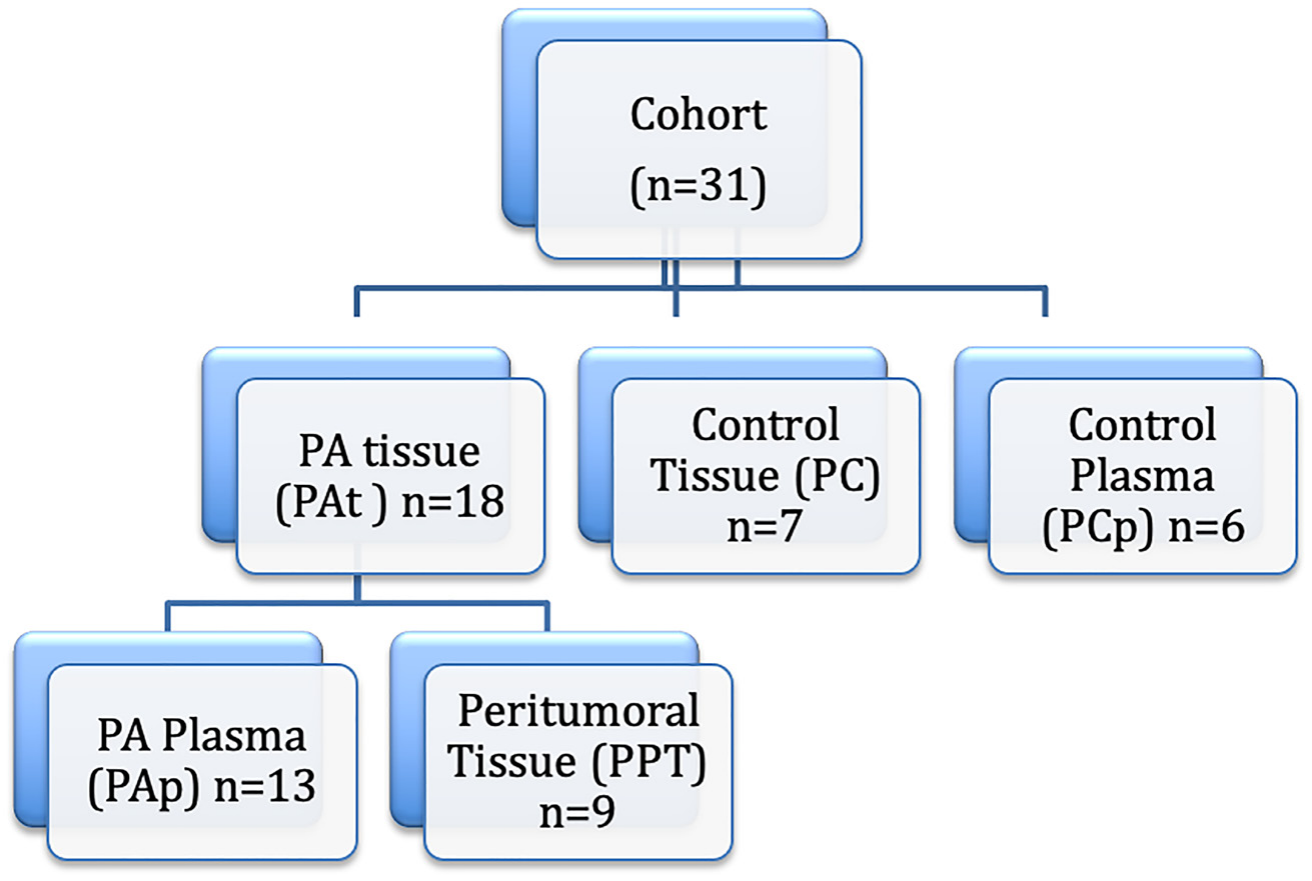
Study design. PA: pancreatic ductal adenocarcinoma.

We studied 18 patients with confirmed tissue diagnosis of PA (PAt group) and no prior therapy for PA. All pancreatic tumor samples were collected during surgery. Part of the tumor tissue was micro dissected by an experienced pathologist to separate tumor from non-tumor tissues. The micro dissected tumor tissue was disrupted using a Polytron^™^ homogenizer and kept at –70°C for molecular studies. The control group consisted of seven normal pancreatic tissue samples (PC group) obtained during surgery from multiple organ donors or pancreatic surgery for trauma (Figure 6).

Twenty ml of blood were collected from patients before surgery and equally distributed into EDTA-coated tubes. Specimens were initially stored at 4–8°C, and then rapidly processed by centrifugation followed by supernatant collection. After processing, all supernatants were stored at −80°C until analysis. In addition, 6 plasma samples from normal controls were used for the determination of plasma miRNAs expression (PCp group) (Figure 6).

Relevant clinical data were collected at the time of the procedure, and the diagnosis of PA required histopathological confirmation. All individuals with PA were enrolled before the initiation of therapy.

### Methods

For tissue analysis, total RNA was isolated with the TRIzol^®^ reagent (Invitrogen Life Technologies, Carlsbad, CA, USA). Sample integrity was evaluated by spectrophotometry at an absorbance of 260/280 nm and by agarose gel electrophoresis. cDNA was obtained using a High-capacity cDNA Reverse Transcription kit (Applied Biosystems, Foster City, CA, USA). For plasma analysis, miRNA was isolated using the miRNeasy kit (QIAGEN, Hilden, Germany), as described previously [53].

Based on previous studies, we assayed plasma, PA tissue and peritumoral tissue for 6 miRNAs (miR-21, -23a, -100, -107, -181c, -210) with a known or suspected association with PA [6, 26–29, 54, 55].

The relative expression of miR-21, miR-23a, miR-100, miR-107, miR-181c and miR-210 and of the endogenous controls RNU-24 and RNU-48 was determined by TaqMan^®^ Real Time PCR Assay (Applied Biosystems, Foster City, CA, USA). miR-16 was used as control for plasma miRNA expression, as previously reported [12, 20]. The specific probes and assay IDs are presented in Supplementary Table 1. Reactions were incubated in a 96-well optical plate at 95°C for 10 min, followed by 40 cycles at 95°C for 15 sec and at 60°C for 1 min. Gene expression was calculated using the QPCR software 40 with the determination of the efficiency of each reaction.

Total RNA was isolated from the samples using the Trizol-LS reagent (Life Technologies, Carlsbad, CA, USA). Complementary DNA was generated using 10 ng of RNA in combination with miRNA-21, -23a, -100, -107, -181c, -210, RNU-24 and RNU-48 reverse transcription primers and a miRNA reverse transcription kit (Life Technologies) according to the manufacturer’s recommendations. Quantitative PCR was performed for each miRNA using Taqman miRNA expression assay reagents (Life Technologies, Grand Island, NY, USA). Expression levels for all candidate miRNAs were normalized to RNU-24 and RNU-48, which was expressed at similar levels in all samples, exhibiting < 1 cycle threshold (Ct) difference across samples. After normalization to RNU-24 and RNU-48 (ΔCt), the ΔCt values for control miRNAs were averaged and subtracted from the ΔCt values of each individual sample (ΔΔCt) and expression levels were calculated by the 2−ΔΔCt method which indicates a twofold difference per every difference in normalized Ct values.

### Statistical analysis

The expression of each miRNA is reported as mean, standard deviation, median, and range. Sample and laboratory data, and miRNA expression levels for the patients in the PA and control groups were compared by analysis of variance for normally distributed continuous variables, by the nonparametric Kruskal–Wallis tests for non-normally distributed continuous variables or by the Mann-Whitney test for categorical variables. Differences between the groups was done using Dunn’s post-test for multiple comparisons, with a significance level of 0.05. Receiver-operating characteristic (ROC) curves and the area under the ROC curve (AUC) were used to assess the feasibility of tissue and plasma miRNAs as a diagnostic tool for detecting PA. Data were analyzed using the R Project Software [32]. Differences were considered significant at *p* < 0.05.

## SUPPLEMENTARY MATERIALS



## Abbreviations

PA: pancreatic ductal adenocarcinoma
Pap: samples of plasma
PAt: pancreatic tumors
PPT: peritumoral regions
PC: control group
PCp: healthy volunteers donated blood
