# Supplementary material for: The role of circulating miRNAs and CA19-9 in pancreatic cancer diagnosis
Source: Oncotarget. 2021 Aug 17;12(17):1638–50. doi: 10.18632/oncotarget.28038 (PMC8378767; doi:10.18632/oncotarget.28038)
Supplement: Supplementary file 1 [file oncotarget-12-1638-s001.pdf]

The role of circulating miRNAs and CA19-9 in pancreatic cancer diagnosis

SUPPLEMENTARY MATERIALS

Supplementary Table 1: miRNAs assays

miRNA	Assay ID
**miR-21**	002438
**miR-23a**	002439
**miR-100**	002142
**miR-107**	000443
**miR-181c**	002333
**miR-210**	000512
**RNU-24**	001001

Assay ID: ID from qPCR Taqman™ (Life Technologies).
